# «Digesting Crohn’s Disease»: The Journey of Young Adults since Diagnosis

**DOI:** 10.3390/jcm12227128

**Published:** 2023-11-16

**Authors:** Nathalie Touma, Louise Zanni, Pierre Blanc, Guillaume Savoye, Carolina Baeza-Velasco

**Affiliations:** 1 Laboratoire de Psychopathologie et Processus de Santé, Université Paris Cité, 92100 Boulogne-Billancourt, France; louise.zanni@u-paris.fr (L.Z.); carolina.baeza-velasco@u-paris.fr (C.B.-V.); 2 Service d’Hépato-Gastroentérologie B, Centre Hospitalier Universitaire de Montpellier, 371 Av. du Doyen Gaston Giraud, 34090 Montpellier, France; p-blanc@chu-montpellier.fr; 3 Service d’Hépato-Gastroentérologie, Centre Hospitalier Universitaire de Rouen, Université de Rouen Normandie, UMR 1073, 76000 Rouen, France; 4 Département d’Urgences et Post-Urgences Psychiatriques, Centre Hospitalier Universitaire de Montpellier, 371 Av. du Doyen Gaston Giraud, 34090 Montpellier, France

**Keywords:** psychosocial adjustment to illness, illness identity, Crohn’s disease, young adults, emerging adults

## Abstract

Crohn’s disease affects 2.5 million people in Europe (more than 100,000 people in France) and often occurs between the ages of 15 and 30, a period marked by self-construction. However, few studies have focused on the experience of the diagnosis during this sensitive developmental stage. This study aimed to qualitatively explore the experience of Crohn’s disease in young adults since their diagnosis. Fifteen young adults (18–35 years) diagnosed with Crohn’s disease participated in a semi-directive interview. Narrative data were subjected to a thematic analysis, and thirty percent of the interviews were double-coded. The results revealed an evolution of four main themes since diagnosis: (1) course of care, (2) illness perceptions, (3) disease management and (4) self-perception. For most participants, the onset of the disease was difficult, marked by severe symptoms requiring hospitalization, numerous medical examinations and sometimes several consultations before diagnosis. This journey was more difficult when it was associated with negative relations with the medical staff, who were sometimes perceived as unsupportive. Thus, some people described this diagnostic period as an “ordeal”, while others experienced it as a “relief” from their suffering. The announcement of the diagnosis was often a “shock”, an “upheaval” or a “downfall”, followed by phases of denial associated with a desire to maintain a “normal life” and not to be defined by the disease. Despite a difficult start, most participants grew from their experience with CD, with a sense of a personal development that was made possible by self-regulation processes that enabled them to draw on their own experience and resources to adjust to their illness. By highlighting positive possibilities for evolution, this study suggests the importance of supporting the psychological resources of young adults by proposing, at an early stage, psychological support or therapies focused on acceptance and engagement.

## 1. Introduction

### 1.1. Crohn’s Disease

Crohn’s disease (CD) is, along with ulcerative colitis, an inflammatory bowel disease (IBD). IBD is a major public health concern, affecting nearly 10 million individuals worldwide and 2.5 million in Europe [1]. According to the French National IBD’s Observatory, in 2019, nearly 200,000 people were affected in France, and more than 100,000 suffered specifically from Crohn’s disease (CD). In 11 years, between 2008 and 2019, there was an increase of more than 75% in cases of IBD among young people aged from 13 to 19 years old [1]. It is, in fact, often declared at a young age, with 20% of pediatric cases and a peak of diagnosis between the ages of 15 and 30 years old.

CD is characterized by inflammation in the gastrointestinal tract leading to abdominal pain, nausea, vomiting and episodes of diarrhea. These symptoms are suggestive of CD if they persist over time and associate with a general health degradation, leading to important weight loss, deficiencies and chronic asthenia [2,3]. Patients can experience complications such as abscesses, fistulas or intestinal obstructions and extra-intestinal manifestations such as joint pain or skin inflammations [4].

To date, there is no curative treatment. The aim is, therefore, to first stop the progression of a flare, then to maintain a state of remission [5,6]. However, it is difficult to predict the response to a treatment, just as it is difficult to foresee the flare frequency [7]. Patients with CD thus face uncertain prospects of recovery and must cope with the unpredictable and chronic aspects of their disease.

### 1.2. Crohn’s Disease Psychosocial Outcomes

Because of its unpredictability, disabling symptoms and low prospects of recovery, patients with CD can face psychopathological and health-related challenges. The literature reveals that 17% to 50% of adults with IBD show high levels of anxiety and depressive symptoms [8,9,10,11,12] and 20% to 74% report an impaired health-related quality of life (HRQoL) [10,13,14,15]. Adults with IBD state a change in their habits to adapt to the unpredictability of symptoms [16,17,18]. Some also report feelings of losing opportunities in life, since IBD interferes with their work and social life [16,17,18].

Qualitative studies reveal that IBD also threatens patients’ self-consciousness and identity, with participants often reporting feeling consumed by their illness [19,20,21,22]. Therefore, in addition to physical, emotional and social repercussions, IBD seems to affect cognitive aspects, altering the way patients perceive themselves [22].

### 1.3. The Specific Case of Young Adults with Crohn’s Disease

The challenge of this period from adolescence to adulthood is the exploration of one’s self and identity, a complex task at this sensitive developmental stage [23,24]. Young people in their 20s (emerging adults between the ages of 18 and 25) are searching for themselves and gradually building the foundations of their adult lives, and those closer to their 30s (young adults between the ages of 26 and 35) have already achieved a certain “stability” but are still at the beginning of their adult lives [23,24].

If the challenge of early adulthood is to achieve a sense of coherence and identity, the challenge for individuals, whatever their age, is to maintain this identity despite change. Indeed, some significant life events can raise personal questioning and sometimes require changes in a person’s perception of themselves and their life. The diagnosis of a disease, especially if it is chronic, can be one of those life-changing events, with its share of upheavals [25]. Thus, young adults with chronic and invalidating conditions can be confronted with a double task: their self-exploration and the adjustment to their illness, which itself can lead to the reconsideration of some aspects of themselves and their life.

Even though CD is often declared at a young age, few studies have focused on emerging and young adults. Yet, some authors note that younger patients and those with a shorter time since diagnosis have worse mental and physical health compared with older patients with a longer time with their disease [10,13,26,27,28].

### 1.4. Identity Challenges in Young Adults with Crohn’s Disease

A few qualitative studies have revealed some identity issues raised by the disease. Most participants report that IBD threatens their self-consciousness and sense of identity [19,20,21,22,29]. Some even report a compromised identity, enhanced by the feeling of being treated differently by their environment [19]. Many of them also mention a feeling of otherness compared with others and compared with themselves before the diagnosis [29]. This feeling seems more important for those diagnosed for less than two years, because they declare feeling more challenged in their identity than those diagnosed for a longer period [20]. If the patients’ declarations essentially reveal a negative impact of the illness, many also mention a more positive dimension. This tendency is usually noticeable among those who have been experiencing their disease for some time and are thus more advanced in their adjustment process. Amongst those interviewed by Purc-Stephenson et al. [17], 73% declared that their IBD had a positive impact on their life. The main developments reported are linked to a personal and a spiritual growth, a better appreciation of their life and new perspectives. Similarly, Wu et al. [30] recently demonstrated post-traumatic growth in adolescents and emerging adults (15–25 years old) with IBD. Their interviews revealed the transition from negative to more positive emotions, a cognitive restructuring, a change in their philosophy and more optimistic perspectives of their future [30].

Patients’ declarations thus uncover that, in addition to its physical, emotional and social repercussions, IBD seems to affect some cognitive aspects, altering the way they perceive themselves. However, the majority of these studies are essentially focused on the adult population and IBD in general. It seems pertinent to focus on younger patients diagnosed with CD, since it has the particularity of being declared at a sensitive developmental stage. Thus, this study aimed to explore qualitatively the experiences of emerging adults and young adults diagnosed with CD. More specifically, our objective was to retrace, through individual semi-structured interviews, their experiences of their disease since their diagnosis by exploring the perceptions they have of themselves, their disease and its impact in their daily life.

## 2. Method

### 2.1. Design, Participants and Procedure

This study is cross-sectional and retrospective and relies on a qualitative method. In order to better understand the singularity of the experience of young adults with CD and, given that the complexity of an experience can only be grasped through the unfolding of a narrative about this lived experience, we chose to conduct and analyze clinical research interviews.

Inclusion criteria for participants were a diagnosis of CD in their adolescence or early adulthood and being aged between 18 and 35 years old. They were recruited between December 2022 and April 2023. The recruitment took place in the gastroenterology departments of “Centre Hospitalier Universitaire de Montpelllier” and “Centre Hospitalier Universitaire de Rouen” in France. This study was proposed by doctors (P.B and G.S) to eligible patients, and an informational note and consent form were handed to those interested. Those who wanted to participate delivered an oral consent authorizing the doctor to transfer their contact to the principal investigator (N.T). Participation was voluntary; those interested were contacted via e-mail or text message to arrange a date for the interview. Their participation was only possible if they transmitted via e-mail, prior to the interview, their written consent and signed audio-recording authorization.

The individual interviews were led by a female research psychologist (N.T), who was not affiliated with any of the recruitment sites and therefore did not meet with the participants prior to the interviews. These were conducted by videoconference via the Zoom application that was hosted by the secure server of the researcher’s institution. Interviews were recorded with a voice recorder dedicated to this study and that was supplied by the principal investigator’s research laboratory. Interviews consisted of 2 stages: the 1st part was structured and aimed to collect clinical and sociodemographic data; it was therefore not recorded. The second part was semi-structured and started with the same open-ended question for all participants *“Can you tell me about your experience of your Crohn’s disease diagnosis, as a person that was diagnosed at a young age?”.* Prompting, rephrasing and follow-up questions encouraged participants to develop and elaborate their expression. Some specific aspects, related to the psychosocial adjustment to the disease in the literature, were addressed if they were not spontaneously mentioned (Table 1). Thus, there was no pre-established fixed grid and the questions were adapted to the participants’ spontaneous speech.

### 2.2. Data Analysis

A thematic analysis was conducted using NVivo, with a license provided by the principal investigator’s institution, since it facilitates advanced data management, analysis and visualization. This inductive method enabled us to identify the most common themes and the most representative of the exchanges, as well as to describe the differences and similarities found to bring out the singularity of the experiences. The thematic analysis was carried out in several stages: (1) in-depth reading of the transcribed interviews, (2) coding of each unit of meaning (known as verbatim), (3) grouping of similar codes into themes, (4) refinement of themes to obtain major themes and sub-themes and finally (5) construction of a thematic tree offering an overview of the main contents discussed and representing the articulation of themes and sub-themes.

To enrich our analyses, part of the corpus was double-coded by another research psychologist, a female doctoral student in psychology (L.Z), who also had no connection with the gastroenterology departments or the participants. We used a sequenced analysis, more suited to a team approach [31]. In fact, sequenced analysis allows two or more researchers to code data independently. By comparing the results of these codings, differences in interpretation or understanding can be identified and discussed to reach a consensus, thereby improving the quality of the results and minimizing the risk of individual bias. This approach can thus lead to in-depth discussions between researchers about the data and possible interpretations, which can foster a richer understanding of the phenomenon under study.

In this sense, each party (N.T and L.Z) first independently analyzed 3 randomly selected interviews, with the aim of creating an initial theme sheet. Comparisons of the themes and discussion of the differences led to an initial grid that was applied to the rest of the interviews, with the limited possibility of themes being added. In a second stage, 30% of the interviews (n = 5) were double-coded using the pre-established grid. The analysis of these 5 independently coded interviews was then compared to identify any new themes or sub-themes that might have emerged and to discuss the relevance of retaining them in the final analysis.

When constructing our qualitative methodology, we relied on the recommendations of Paillé and Mucchielli [31]. When developing our thematic analysis approach, we referred to the recommendations of Braun and Clark [32], who advocate a reflexive analysis rather than one based on coding reliability. An approach based on coding reliability favors precision through the use of a structured codebook established by several raters, followed by the calculation of inter-rater reliability. The use of such scores suggests that appropriate techniques can achieve the same analysis. However, qualitative methodology is intrinsically imbued with the subjectivity of the researcher, so there is no single “right” way to code data. Braun and Clark [32] therefore suggest a more flexible approach to coding. They see thematic analysis as an active, evolving and reflexive process. With this in mind, our double-coded analysis was not intended to verify the inter-rater fidelity of our analyses but rather to enable constructive exchanges and reflection on the analysis of the interviews. Finally, to write this article, we followed the consolidated criteria for reporting qualitative research checklist (COREQ) (Table S1) [33].

### 2.3. Ethical Considerations

This study has been approved by the Research Ethics Committee of Université Paris Cité (N° IRB: 00012022-123). The project’s compliance with Reference Methodology 004 (MR-004) of the General Data Protection Regulation concerning research not involving the human person has been verified and confirmed by Université Paris Cité’s data protection officer. This research has therefore been carried out in compliance with the legislative and regulatory provisions in force. All participants signed an informed consent that was transmitted via e-mail to the principal investigator (N.T) prior to the interview.

In order to respect participants’ confidentiality, all interview data were non-nominative. Socio-demographic and clinical data were collected before recording began, and participants were reminded of the importance of avoiding mentioning personal data such as surnames, first names, cities or hospitals during the recorded interview. If a participant inadvertently mentioned sensitive data, they were omitted or anonymized during transcription. A numerical code corresponding to the order of recruitment was assigned to each participant in order to cross-reference their transcribed interview with their clinical and socio-demographic data collected on a password-protected Excel file on a password-protected laboratory laptop.

## 3. Results

### 3.1. Participant Characteristics

This study was proposed to 26 eligible patients who were interested in the research. A total of 19 interviews were conducted and we reached theme saturation on the 15th interview, which meant that after this no new information or themes emerged from the data. More specifically, the five main themes emerged very early on in the analyses after coding the first three interviews. The saturation of sub-themes, however, was reached after the 13th interview was coded, since no additional themes or sub-themes were added to the thematic list. The principal investigator (N.T) went on to code two further interviews to confirm this observation. Following a discussion with the second investigator (who double coded 30% of the interviews (L.Z)), the authors concluded that thematic saturation had been reached. This research thus followed an inductive thematic saturation model, which focuses on the analysis and more specifically on the emergence of new codes or themes [34].

Interviews lasted between 23 and 75 min, with a mean duration of 53 min. Participants were aged between 20 and 37 years old at the time of the interviews, with a mean age of 29.3 years (SD = 5.1). Amongst the 15 participants, 60% were female and 67% were in a couple or married. Furthermore, 33% had a master’s degree and 93% were professionally active.

Regarding clinical data, all participants had an established CD diagnosis, as they were recruited in hospitals by their doctor. They were diagnosed between the ages of 14 and 33 years old, with a mean age of 18 years old at the onset of first symptoms (SD = 4.9), a mean age of 19 years old at diagnosis (SD = 4.9) and a mean duration of 9.9 years since diagnosis (SD = 5.6). A large majority (87%) considered their disease stabilized by treatments, which meant that some flares were possible occasionally, randomly or spaced-out over time. All clinical and socio-demographic data collected are presented in Table 2.

### 3.2. Thematic Analysis of Major Themes

**Figure 1:**
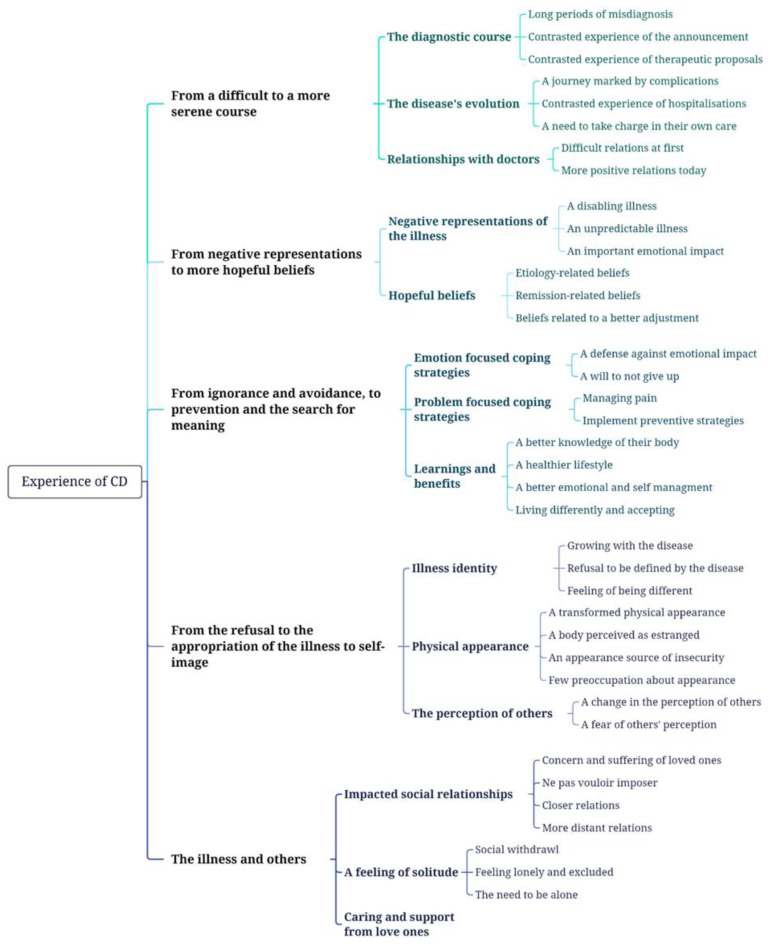
Thematic tree.

The thematic analysis revealed five themes linked to: (1) the disease’s course, (2) illness perceptions, (3) disease management, (4) self-perception with the illness and (5) social repercussions. In this article, we chose to develop the first four themes that answered our research question and focused on the processes of adjustment to illness. Themes and sub-themes are illustrated in the thematic tree below (Figure 1).

#### 3.2.1. The Disease Course: From a Difficult Course to a More Serene Pathway

Most participants reported a late diagnosis, with a long and difficult wait (67%). This wandering prolonged the pain of participants and reinforced their confusion and concern regarding their health status. Even though the diagnosis was usually expected, participants expressed mixed feelings after their CD diagnosis. The diagnosis led to new questions (80%) and apprehensions that threatened their prospects (80%).

*“The first thing I wondered about was the impact it would have later on [...] and whether I was going to die sooner than if I hadn’t had this disease [...] all these worries about death, about the disease getting worse, that was really the first thing that came in mind”*

(Ms. S, 23 years old)

The moment of diagnosis was therefore difficult for most participants, who characterized it as a “shock”, with the impression of falling or being struck, which attested to the violence felt at that moment (73%). The rupture caused by the announcement led to phases of denial to defend themselves against a reality that was difficult to accept (60%).

*“It’s the biological, clinical pain of having a complicated disease that alters your quality of life, and it’s also the moral, psychological pain, not to say the trauma, because you are condemned”*

(Ms. F, 37 years old)

*“I was in a state of denial because I was still digesting this disease”*

(Ms. L, 31 years old)

Even though, for a majority of participants, the diagnosis announcement was essentially difficult, for some, the shock coexisted with relief and the hope of identifying a treatment that could finally relieve their pain (53%).

*“It reassured me to know what I had, that it was explainable, that there was a treatment and that I would be OK”*

(Mr. J, 29 years old)

However, many participants also reported mixed feelings about treatment, with fears and hesitations in the face of protocols that were not always immediately efficient (67%). In fact, the majority stated a course marked by a recurrence of flares and/or severe complications (80%). These aggravations were morally difficult for the participants, who expressed a weariness in the face of a pathology that did not seem to stabilize despite their observance of treatments.

*“I’d been in and out of hospital several times [...] I had to undergo emergency surgery and had 21 cm of intestine removed [...] the intestine was really rotten and sticking to the abdominal wall. After that, I had a lot of problems [...] crisis after crisis, lots of intestinal occlusions. Last year I had maybe 7 intestinal obstructions, and each time I had to stay in hospital for 4–5 days”.*

(Mr. N, 30 years old)

The hospitalizations and the related care were also physically (33%) and mentally (87%) exhausting. Participants described themselves as “out of energy” and “out of breath”. A majority also reported a feeling of resignation and an impression of having no choice but to endure their doctors’ decisions (73%). For some, however, the discomfort that could be caused by care coexisted with a relief of seeing their symptoms improve (53%).

*“The hospital was a gloomy environment, I swear it was very hard because we leave feeling down and completely drained”*

(Ms. F, 37 years old)

Participants’ experiences of the disease were nonetheless influenced by the quality of their relationship with the professionals in charge of their care. A majority reported negative initial experiences with the first doctors they consulted, especially at the time of diagnosis (80%). However, many reported more positive relations with their actual doctors, allowing more serenity regarding their ongoing care (87%).

*“The doctor who took care of me at the beginning tended to treat me like I was just a “patient file”, I wasn’t a human being”.*

(Ms. S, 37 years old)

*“I have the cell phone number of the Professor, I know I can reach him if I need it […]. I know I can count on the medical staff, so it reassures me”*

(Ms. J, 28 years old)

#### 3.2.2. Illness Perceptions: From Negative Representations to More Hopeful Beliefs

The diagnosis announcement led to attempts to make sense of their experience with CD. The disease was described as restrictive and disabling by a large majority of participants (93%). The restrictions were mainly linked to the symptoms that significantly interfered with their daily lives, especially during flares. Abdominal pain was the main complaint, often described as “excruciating” and “violent”. Participants reported a body drained of energy that had no more physical or psychological strength, even when the disease was in remission.

*“I was very tired, even today […]. No matter how much I slept or didn’t sleep, I was tired. So sometimes I don’t want to do anything […]. Fatigue is really like a leitmotif, it’s recurrent. It really bothered me a lot, it’s tiring in fact, it’s something that’s very tiring daily”*

(Ms. M, 28 years old)

The gastrointestinal symptoms, especially diarrhea, were also particularly restrictive and interfered with their daily activities. Some also reported humiliating episodes of incontinence.

*“I couldn’t hold myself, to the point that one day I was about to take the metro and there was a 4–5 min wait and I told myself that I would never make it. So, I got out of the metro and went between two cars. It was very humiliating, even though no one saw me, but I told myself it is not possible I am not capable of holding myself. It lasted for months, it was very complicated to manage personally”*

(Ms. M, 28 years old)

In addition to symptoms, some consequences of the disease also imposed limitations. A majority essentially reported diets that they experienced as frustrating, especially during moments of conviviality with family and friends.

*“The disease changed my relationship with food. I like to drink, I like to eat, I also like to smoke and all the bad good thing in life were taken away from me […]. These were the three things that I would allow myself to escape daily life and to erase this, it was a big constraint”*

(Ms. S, 37 years old)

Moreover, CD’s constraints sometimes led to certain renouncements. Whether it was on a social, family, personal or professional level, some people felt they were losing life opportunities and losing their freedom, with the impression they were no longer living because of the disease.

*“It is heavy when we are young because we want to do a lot of things […] we rather think about having fun, going out, pursuing studies, work and not necessarily live with this disease”*

(Mr. L, 34 years old)

Many of them reported an important emotional impact and a more sensitive mood, with more depressive, irritable and/or anxious tendencies (60%). The restrictions linked to the symptoms were often amplified by the unpredictable aspect of CD. This unpredictability led to the constant anticipation of the symptoms’ resurgence, which did not allow them to peacefully apprehend their daily life, even when the disease was in remission.

*“It is a recurrent anxiety […] when I had my oral or written exams I used to wonder: what if it doesn’t go well? If I am suddenly in pain, what do I do?”*

(Ms. F, 37 years old)

Although their illness representations were essentially negative, their beliefs on their illness evolution suggested the hope of a “cure”. In an attempt to make sense of their experience, participants elaborated on theories regarding the illness’ etiology and the factors influencing remission. For some, it was their lifestyle that could explain the declaration of their CD and the resurgence of a flare (46%). Therefore, many of them believed that the maintenance of a healthier lifestyle could allow the stabilization of their illness (40%); quitting smoking and watching their diet were the most frequently reported.

*“I was able to stabilize the disease by doing several things […] note what I eat, favor raw over cooked foods, cook rather than bye processed meals and of course I quit smoking […] and overall pay more attention to my lifestyle”*

(Ms. L, 31 years old)

Even though conventional treatments were perceived as lifesaving when they were efficient, some participants reported a better efficacity of alternative treatments such as naturopathy (13%). Many of them also mentioned psychological causes (53%). Some even reported the image of a body that “speaks” through the illness, a flare being interpreted as a sign of an emotional burden that was denied and that was therefore expressing itself through the body. Thus, for some participants, a better knowledge of the disease’s emotional impact and the solicitation of psychological support allowed a better adjustment (47%).

*“I know that when I am in a negative phase emotionally, after a traumatizing life-event such as a separation or the loss of someone close, I know that the disease will be more present. I also know I will be more in pain when I am upset or angry, I know it is going to make the disease worse”*

(Mr. L, 34 years old)

#### 3.2.3. Managing the Disease: From Ignorance and Avoidance to Prevention and the Search for Meaning

Many participants reported phases of trial and error in the early stages to identify the strategies that worked best for them in dealing with symptoms, thus describing a learning process. At first, many participants reported ignorance of symptoms (73%). For some, it was a way to defend themselves from a reality that was difficult to accept, for others it was the reflection of a committed battle or even rebelling against the disease (33%). However, this permanent struggle sometime led to a weariness, taking it upon one’s self becoming more a form of resignation.

*“When I was in pain, I took it upon myself. It was the beginning, I was fighting”*

(Mr. J, 28 years old)

For some participants, ignorance allowed them to temporarily maintain the illusion of a “normal life”, for others the disease took over their life from the start. Many of them reported they preferred to avoid certain situations to reduce their anxiety in the face of the disease’s unpredictability (27%), which procured them a feeling of security and control. Avoidance was, however, generally a temporary coping strategy, since it often led to a feeling of lost freedom.

*“I had two choices, either I condemned myself to not living and let the disease take over my life, or I took control of the disease and lived. So, in the beginning I preferred to restrict myself to everything, but as time goes by you tell yourself that you’re young, that you’ve got your whole life ahead of you and that you’ve got to get on top of it”*

(Ms. J, 28 years old)

It was time and experience that allowed participants to consider more effective strategies. Breathing and meditation techniques were sometimes reported, allowing them to re-mobilize internally for a better management of their pain (41%). For some, it was particularly yoga or sophrology that allowed them to reconnect with their emotions and physical sensations.

*“When pain is intense, I try to take time to breath […] I focus on the pain to try to reduce it”*

(Mr. N, 30 years old)

Having successfully overcome certain challenges, many participants became aware of their resources. Some reported a more positive and optimistic attitude, with a feeling of taking over their illness (53%).

*“We try to hold on to the positive inside the negative. I think that it plays an important role, I really think a have a mental strength”*

(Mr. J, 28 years old)

Although emotional management was particularly emphasized by participants, many of them also reported pain management strategies, especially during flares. The use of painkillers and the identification of what soothed their symptoms were often mentioned (60%), but when the pain became unbearable, going to the emergency room was sometimes perceived as the only option (20%). However, many of them stated they were today more able to consider more preventive strategies that they permanently put in place even in periods of remission to prevent the aggravation of symptoms (93%). They had adapted their daily lives to better adjust to their disease. The most frequently mentioned improvements were rebalancing their diet and checking toilet access.

*“I planned everything. For example, in my bag I’d pack toilet paper and wipes. If I went to such and such a place, I’d make sure there was a toilet nearby, or a McDonald’s where I could get to the toilet. In fact, everything was planned, every outing was planned”*

(Ms. J, 28 years old)

Many of them felt they were better armed today to anticipate their needs. Most of them had assimilated their adjustment process to real personal growth, since their journey has led to life lessons and secondary benefits. They reported a better knowledge about their illness, their body (80%), themselves and their emotions (33%), which allowed them to accept their situation and live differently with their illness (73%).

*“Even if this illness was complicated to manage, it brought me a lot of positive things […]. I am realizing that since this illness I seen life differently […] I live every moment more intensely”*

(Ms. C, 25 years old)

#### 3.2.4. Illness Identity: From Refusal to the Appropriation of the Disease to Self-Image

The illness seems to have led to certain identity redefinitions, since many of them reported an evolution in their self-perception since diagnosis. The changes imposed by the illness requestioned an already ongoing self-construction (73%).

*“The illness came at a moment when adolescence is taking place, when puberty is taking place, so I had a hard time with that”*

(Mr. K, 26 years old)

Many of them mentioned a rupture in the image they had of themselves. Thus, some refused to be defined by the illness or to be categorized as being sick in an attempt to defend themselves and maintain a certain normality (80%). This refusal was sometimes linked to the negative representations they had of a “sick person”. In fact, their statements revealed a discrepancy between the way they saw themselves and the way they saw a sick person (who was very much associated with old age and disability). Furthermore, the remissions that were possible during their course did not allow them to identify with the image of an ill person since their illness was not always present.

*“Many people told me to ask for the disabled status, but psychologically I couldn’t do it. I tell myself I am not disabled. In addition, it is a status you keep for life, you cannot one day no longer be disabled. I tell myself that one day I will no longer have this disease”*

(Ms. C, 25 years old)

If the majority refused to perceive themselves as ill, more than half of the participants reported feeling different since their diagnosis (60%). Some felt changed compared with themselves from before, revealing a gap between who they were today and who they were before the illness. Their representations were essentially negative, with a feeling of being more fragile and vulnerable and sometimes with the impression of being transformed physically and psychologically. Others also felt different compared with others, with the impression that the illness did not allow them to evolve like their peers.

*“Before I was a very lively person, but then I felt transformed, I was like a dragon, nobody could talk to me”*

(Ms. S, 37 years old)

Many of them also reported the feeling of a transformed appearance (80%) and a body that was sometimes perceived as estranged (40%). It was essentially their weight fluctuation that was often mentioned and difficult to accept, since it was the physical sign of a “sick”, weak and failing body. The image of a “skeletal” body, eaten away by the disease, with only “skin and bones”, came up frequently in their statements, suggesting a perception that was more associated to death rather than life. Some even reported difficulty in identifying and recognizing themselves in this new image.

*“Yes, I was skeletal, and I was hunched over because of the operation, because of the scar, I had trouble straightening up. So yes, it was weird to see myself like that and it was hard to accept. When I saw myself, I didn’t recognize myself anymore”*

(Mr. N, 30 years old)

Physical changes seemed to be a source of insecurity, especially in social relationships and particularly in first romantic experiences (36%). Many of them reported a loss of self-confidence and self-esteem. These physical and identity challenges sometimes led to feelings of stigmatization, shame and a desire to hide their illness (87%).

*“I was afraid that no one would accept it, that people would think I was sick. I was really afraid it would be repulsive”.*

(Ms. J, 28 years old)

While, for some, these feelings persist to this day, others spoke of an evolution towards a more positive self-perception and an acceptance of their illness as part of themselves without it defining them.

*“Illness is not our whole life, it’s a painful part of it, but it’s not our whole life at all”.*

(Ms. S, 23 years old)

## 4. Discussion

The themes that emerged in our analyses converge with those reported by several qualitative studies focused on adolescence or adulthood that also revealed the repercussions that CD could have on the physical, psychological and social quality of life [19,20,21,22]. However, a specificity of our results on young adults was the identity challenges and concerns about physical transformations that our analyses revealed and that were not so prevalent in the adult population. Moreover, if all previous studies reported the negative repercussions and challenges that patients can face and, even though two studies revealed the presence of positive outcomes in IBD [17,30], the interviews that we conducted highlighted the evolutions in the adjustment to CD and more specifically the process by which French young adults evolved positively despite the challenges and how they integrated their illness with their identity.

More specifically, our analyses suggest that it was essentially a process of “self-regulation” of the illness, as described by Leventhal et al. [35], that could explain the positive evolution in young adults’ adjustment. Their initial, rather negative, representations of the disease gave way to emotional strategies aimed initially at preserving them from this threat and remobilizing them internally. The evolution of their experience with the disease, however, led to a re-evaluation of the strategies and representations they had built up, which gradually evolved towards more positive perceptions and thus led to a reconsideration of their adjustment to the disease [35]. Participants seemed to have drawn on their own resources to adjust to their illness, which led to positive evolutions. In fact, similarly to previous findings [30], their adjustment process seemed to allow a post-traumatic growth [36,37]. The diagnosis of CD appeared to create a real rupture in their daily lives through the limitations and changes it imposed. Confrontation with CD certainly gave rise to negative thoughts and emotions, but these seemed to reflect a process of elaboration and an attempt to apprehend and integrate the event into their history. This confrontation prompted most participants to question their way of life and to reconsider what was conceivable for them before the illness but was no longer so today [36]. For instance, the phases of denial and avoidance seemed to initially preserve them emotionally from the shock of the announcement and its repercussions [38,39]. However, these strategies gradually gave way to a more constructive process, enabling several participants to revisit their representations and resources. Over time, we thus observed the implementation of more proactive coping strategies, such as symptom prevention and adapting to daily life, but also attempts to make sense of their experience with their illness. The participants who succeeded in achieving this outcome reported a feeling of having evolved, grown and overcome something beyond themselves, with the impression of being better armed in the face of adversity [36,37]. Many of them mentioned having learned from their illness, with a feeling of knowing themselves better, of managing their emotions better and, above all, of having acquired a different way of living. They now reported an evolution towards a more positive self-perception and the acceptance of their illness as part of them without it totally defining them.

All these developments suggest not only an acceptance of this new reality, but above all an appropriation of CD in their life course, with processes similar to those described by Oris et al. [40,41] in their model of illness identity. These authors explored the adjustment to diabetes in adolescents and emerging adults [40]. Their work allowed them to develop a questionnaire that evaluated four possible outcomes of a person’s attempt to integrate illness with their identity [40]. The first two dimensions, engulfment and rejection, refer to difficulty in illness integration. More specifically, engulfment characterizes individuals who tend to define themselves exclusively by their disease, and thus indicate that all domains of their life and their identity are invaded by the illness [40]. Rejection characterizes those who perceive the illness as a threat to their integrity and thus refuse to consider it as part of their identity [40]. In contrast, the two other dimensions refer to more adaptative illness integration. Acceptance characterizes those who accept their illness as part of their identity amongst other assets, and thus try to adjust and adapt their lives with the disease [40]. Enrichment refers to the positive life changes that result from the illness. It characterizes those who undergo personal growth when adjusting to their illness, allowing changes in their perspectives, their priorities and, overall, in their perceptions of themselves and their life [40]. Our participants’ journeys with CD seemed to reflect these processes. After diagnosis, there seemed to be difficulty in integrating the disease with their self-image, which was manifested by their rejection and a feeling of being overwhelmed by the disease. We then noted a progressive appropriation of the diagnosis, with positive changes, the acceptance of their new reality and, for some, a real personal development.

Although this study sheds some light on adjustment to CD in young adults, the cross-sectional and retrospective aspect is an important limitation to consider. Although the participants were able to easily retrace their journey with the disease, it is possible that certain elements escaped their memory. A longitudinal approach would be more relevant to observe the evolution of adjustment to the disease over time. Moreover, most of the participants were diagnosed in their late teens, but two participants were diagnosed at a much younger age, and their testimonies sometimes seemed to indicate a course that would have been different from those diagnosed later. Furthermore, only one participant had a parent with CD, and his confrontation with the disease during his childhood seemed to have led to a different experience with his illness. It thus seems pertinent to further explore the journey of some specific profiles, such as those diagnosed during their childhood, or those who were confronted with the disease prior to their diagnosis.

Despite these limitations, our results shed light on certain clinical perspectives, in particular the value of psychological support from the moment of diagnosis to support resilience and growth capacities from the outset. In fact, very few participants mentioned the suggestion of psychological follow-up or referral to a psychologist following their diagnosis. However, these results also offer us certain research perspectives. Many of them suggest a psychological etiology of their illness and wonder about the impact of their life events and emotions on their physical health. Although some studies have identified the role played by psychosocial factors on adjustment to CD [42,43,44,45], we are not aware of any studies that have explored the link between significant life events and mental health on the onset of the disease or the flare-up of symptoms. These aspects should be explored in further research.

## 5. Conclusions

The testimonials we gathered highlight the possible threat that the diagnosis of CD can represent at this sensitive time of life, especially for the first years after the diagnosis. However, although identity challenges and difficulties in adjustment to the disease were reported, these aspects tend to be referred to in the past tense. Most of the participants grew from their experience with CD, with a sense of a personal development that was made possible by self-regulation processes that enabled them to draw on their own experience and resources to adjust to their illness and integrate it with their identity. By highlighting possible difficulties, but also positive possibilities for evolution, this study suggests the importance of supporting the psychological resources for young adults. These results also lead us to wonder about acceptance and commitment therapy (ACT), since it promotes flexibility by working on acceptance of what is uncontrollable and commitment to actions that improve and enrich quality of life. Even though we are not aware of studies that have explored the benefits of this intervention in a population with IBD, mindfulness-based therapy, on which ACT partly relies, has already proved its effectiveness in the management of IBD [46]. Moreover, a recent study trialed a brief two-session ACT telehealth intervention and showed that half of the participants experienced reduced stress and increased engagement in valued actions [47]. This shows the pertinence of considering ACT to support young adults’ growth capacities in the face of CD.

## Figures and Tables

**Table 1:** General question and topics addressed.

**General Question**
Can you tell me about your experience of being diagnosed with Crohn’s disease as a young person?
**Addressed topics**
What the disease has changed Difficulties or concerns caused by the disease Strategies for coping with illness/symptoms Body/self-image Family relations Friendships Couple/sexual relationships Academic/professional sphere

**Table 2:** Socio-demographic and clinical characteristics of study population.

ID	Age	Sex	Marital Status	Latest Degree	Professional Status	Age at 1st Symptoms	Age at Diagnostic	Reported Disease State	Interview Duration
P1	31	F	Single	BTEC	Self-employed	25	26	Remission	60
P2	29	M	Single	Bachelor	Employee	14	15	Stabilized	46
P3	28	M	Relationship	Master’s	Employee	16	16	Stabilized	49
P4	28	F	Married	Master’s	Employee	17	17	Stabilized	47
P5	26	M	Single	Master’s	Employee	15	17	Stabilized	75
P6	30	M	Married	BTEC	Employee	11	14	Stabilized	60
P7	28	F	Married	Bachelor	Employee	18	18	Stabilized	43
P9	20	F	Single	A levels	Student	18	18	Stabilized	59
P11	25	F	Relationship	Master’s	Unemployed	18	18	Stabilized	41
P15	37	F	Relationship	BTEC	Self-employed	16	24	Stabilized	47
P16	37	F	Relationship	PhD	Self-employed	18	19	Remission	66
P18	24	F	Relationship	Master’s	Employee	17	17	Stabilized	63
P22	25	F	Relationship	A levels	Self-employed	20	21	Stabilized	61
P24	34	M	Relationship	A levels	Self-employed	32	33	Stabilized	23

## Data Availability

The data presented in this study are available on request from the corresponding author. The data are not publicly available due to privacy and ethical restrictions.

## Supplementary Materials

The following supporting information can be downloaded at: https://www.mdpi.com/article/10.3390/jcm12227128/s1, Table S1. COREQ checklist (consolidated criteria for reporting qualitative research).
