# Supplementary material for: «Digesting Crohn’s Disease»: The Journey of Young Adults since Diagnosis
Source: J Clin Med. 2023 Nov 16;12(22):7128. doi: 10.3390/jcm12227128 (PMC10672720; doi:10.3390/jcm12227128)
Supplement: Supplementary file 1 [file jcm-12-07128-s001.zip › jcm-2682653-supplementary.pdf]

Table S1. COREQ checklist (Consolidated criteria for reporting qualitative research).

N° Item	Guide questions/description	Page (explanation)
Domain 1: Research team and reflexivity		
Personal Characteristics		
1. Interviewer/facilitator	Which author/s conducted the interview or focus group?	3
2. Credentials	What were the researcher's credentials?	3
3. Occupation	What was their occupation at the time of the study?	3
4. Gender	Was the researcher male or female?	3
5. Experience and training	What experience or training did the researcher have?	3
Relationship with participants		
6. Relationship established	Was a relationship established prior to study commencement?	3
7. Participant knowledge of the interviewer.	What did the participants know about the researcher? e.g. personal goals, reasons for doing the research	3
8. Interviewer characteristics	What characteristics were reported about the interviewer/facilitator?	3
Domain 2: study design		
Theoretical framework		
9. Methodological orientation and Theory	What methodological orientation was stated to underpin the study?	5
Participant selection		
10. Sampling	How were participants selected?	3, 5
11. Method of approach	How were participants approached?	3
12. Sample size	How many participants were in the study?	5
13. Non-participation	How many people refused to participate or dropped out? Reasons?	5
Setting		
14. Setting of data collection	Where was the data collected?	3
15. Presence of non-participants	Was anyone else present besides the participants and researchers?	No
16. Description of sample	What are the important characteristics of the sample?	5, 6, Table 2
Data collection		
17. Interview guide	Were questions, prompts, guides provided by the authors? Was it pilot tested?	4, Table 1
18. Repeat interviews	Were repeat interviews carried out? If yes, how many?	No
19. Audio/visual recording	Did the research use audio or visual recording to collect the data?	Yes, page 3
20. Field notes	Were field notes made during and/or after the interview or focus group?	No
21. Duration	What was the duration of the interviews or focus group?	23 - 75 min, page 5-6
22. Data saturation	Was data saturation discussed?	5
23. Transcripts returned	Were transcripts returned to participants for comment and/or correction?	No
Domain 3: analysis and findings		
Data analysis		
24. Number of data coders	How many data coders coded the data?	2, pages 4-5
25. Description of the coding tree	Did authors provide a description of the coding tree?	7-13, Figure 1
26. Derivation of themes	Were themes identified in advance or derived from the data?	Derived from data, p3-4
27. Software	What software, if applicable, was used to manage the data?	Nvivo, page 4
28. Participant checking	Did participants provide feedback on the findings?	No
Reporting		
29. Quotations presented	Were participant quotations presented to illustrate the themes / findings? Was each quotation identified?	Yes, pages 7-12
30. Data and findings consistent	Was there consistency between the data presented and the findings?	12-13
31. Clarity of major themes	Were major themes clearly presented in the findings?	7-12, Figure 1
32. Clarity of minor themes	Is there a description of diverse cases or discussion of minor themes?	7-13

Reference:

1. Tong A, Sainsbury P, Craig J (2007) Consolidated criteria for reporting qualitative research (COREQ): a 32-item checklist for interviews and focus groups. Int J Qual Health Care 19: 349-357.
